# Orthogonal Analysis on Mechanical Properties of Basalt–Polypropylene Fiber Mortar

**DOI:** 10.3390/ma13132937

**Published:** 2020-06-30

**Authors:** Huimin Chen, Chunyan Xie, Chao Fu, Jing Liu, Xiuli Wei, Dake Wu

**Affiliations:** 1 College of Engineering and Technology, Southwest University, Chongqing 400715, China; Chenhhhmmm@163.com (H.C.); swauyan@swu.edu.cn (C.X.); fcswu2018@163.com (C.F.); jingliu97@163.com (J.L.); 2 Chongqing Academy of Agricultural Sciences, Chongqing 401329, China; weixiuli.cau@126.com

**Keywords:** basalt fiber, polypropylene fiber, orthogonal test, strength

## Abstract

Orthogonal test method was applied to analyze the strength properties of basalt-polypropylene mortar. The effect of basalt fiber length, polypropylene fiber length, basalt fiber volume content and polypropylene fiber volume content on the 28 d cube compressive strength and flexural strength were investigated. Test results show that comparing with flexural strength, the influence of basalt fiber length and polypropylene fiber length on compressive strength of mortar was greater than on flexural strength. The length of polypropylene fibers contributes the highest to the flexural strength. The effect of basalt fiber on mortar strength is the largest with 6 mm length and 4% content. Polypropylene fiber length has the greatest influence on the compressive strength of fiber mortar, followed by basalt fiber volume content. Volume content of polypropylene fiber has the greatest influence on flexural strength of fiber mortar, followed by polypropylene fiber length. According to the scoring of the efficacy coefficient method, the best ratio combination for compressive and flexural strength was the basalt fiber length of 9 mm, polypropylene fiber length of 6 mm, basalt fiber volume content of 4% and polypropylene fiber volume content of 4%. Compared with the blank samples, the 28 d compressive strength and 28 d flexural strength of the cement mortar samples were increased by 27.4% and 49% respectively. According to the test results, the properties of the fiber were analyzed and evaluated and the mechanism of fiber action and fiber microstructure were analyzed.

## 1. Introduction

Cement matrix composite is a kind of engineering material with cement as the cementing material [1]. The whole destruction process of cement matrix composite runs through different stages and levels and the internal cracks will expand into macro cracks over time, eventually leading to the failure. In view of this damage process, different lengths and different types of fibers can be added to cement matrix composite to inhibit the development of cracks at corresponding stages and levels, so as to improve the mechanical properties of cement matrix composite [2,3,4].

Adding fiber into the matrix is an effective means to obviously improve the mechanical properties of cement matrix composite, especially the toughness. Therefore, a large number of scholars are paying increasing attention to fiber-reinforced cement matrix composite. Most of the research is aimed at a single variety or a single length of fiber but this can only focus on the enhancement of a certain performance [5,6,7,8]. Reasonable addition of fibers of different lengths or varieties can inhibit the destruction of cement matrix composite layer by layer and further strengthen the mechanical properties of cement matrix composite on multiple scales [9,10,11,12].

In the research of hybrid fiber cement matrix composite, the most common one is mixing steel fiber and polypropylene fiber [13,14,15,16]. However, the steel fiber is easy to agglomerate in the mixing process, resulting in uneven distribution in the matrix material, which will affect all aspects of the performance of the matrix material and the high production cost of steel fiber will consume a large amount of steel [17,18]. Basalt fiber as a kind of green environmental protection material, not only has the advantages of low price and wide source of raw materials, high temperature, cold and heat resistance but also has the advantages of strong acid and alkali resistance, excellent insulation, excellent aging resistance and so forth, which can be well applied to practical projects [19,20]. Therefore, in order to accelerate the engineering application of this new composite material, it is particularly urgent to conduct in-depth experimental research and theoretical discussion on basalt- polypropylene hybrid fiber cement matrix composite.

The key to mixing fibers is the size and content of each fiber. Short fiber has a greater effect on strengthening concrete, while long fibers play a greater role in strengthening concrete toughness [21]. The content and length of basalt fiber have certain influence on the mechanical properties of basalt fiber concrete. The results show that the addition of basalt fiber improves the tensile strength of concrete but the compressive strength changes little, while the addition of polypropylene fiber greatly improves its mechanical properties [22,23]. When basalt fiber and polypropylene fiber are added at the same time, the pore structure inside the concrete will also change and the air content will decrease first and then increase with the increase of fiber content, thus the strength will also change [24,25]. By testing the compressive strength, splitting tensile strength and bonding strength of concrete mixed with fiber can obtain the best fiber content and ratio but it is not sure which factor has the greatest influence on the concrete strength [26].

In this paper, the effect of basalt-polypropylene fiber on the 28 d performance of cement mortar was studied. Through orthogonal experiments, the influences of basalt fiber length, polypropylene fiber length, basalt fiber volume content and polypropylene fiber volume content on the compressive and flexural strength of cement mortar were respectively studied. The maximum influence unit of each influence factor on different indexes and the matching combination when the mechanical properties reach the optimum are obtained. Finally, the causes of fiber reinforced cement-based materials were further analyzed through fiber action mechanism and microscopic mechanism analysis.

## 2. Materials and Methods 

### 2.1. Materials

The cement adopts ordinary Portland cement with strength grade of 42.5 and its quality meets the requirements of the current national standard GB175-2007 “General Portland Cement”. The sand was common river sand with fineness modulus of 2.36. The mixing water was tap water from Chongqing area, which meets the quality requirement of water in the specification. The water-reducing agent was a polycarboxylic acid water-reducing agent with a water reducing rate of 26% produced by Shanghai Chenqi chemical technology co., Ltd. (Shanghai, China). The test fibers were chopped basalt fibers produced by Haining Anjie Compound Material Co., Ltd. (Jiaxing, China) and bundled monofilament polypropylene fibers produced by Shanghai Chenqi Chemical Technology Co., Ltd. The physical and mechanical properties of the two fibers were shown in Table 1 and Table 2.

### 2.2. Orthogonal Experimental Design

In order to explore the influence of basalt fiber length (A), polypropylene fiber length (B), basalt fiber volume content (C) and polypropylene fiber volume content (D) on the mechanical properties of cement mortar, the experiment adopts orthogonal test method and the factor-level was shown in Table 3.

Orthogonal experiment was designed according to the selected factor-level and L16(4^5^) orthogonal table was selected, as shown in Table 4.

### 2.3. Specimen Production

The test was divided into two blank groups and two test groups, which were used to measure the compressive strength and flexural strength of the test pieces respectively. According to the GB175-2007 standard, the compressive strength test adopts a cube test block of 70.7 mm × 70.7 mm × 70.7 mm and the flexural strength test adopts a cuboid test block of 40 mm × 40 mm × 160 mm. In the blank group, the cement-sand ratio was 1:3, the water cement ratio was 0.4 and the dosage of water reducing agent was 1% of the cement mass. On the basis of the blank group, the experimental group added basalt fiber and polypropylene fiber in a certain volume.

The mixing sequence of fibers has a great influence on the mechanical properties of mortar, so the mixing sequence used in the test was to mix sand, cement and fiber dry for 2 min, then water was added and mixed for 2 min, so that fibers are evenly dispersed in mortar test blocks, then mixed mortar was put into a mold and the compressive and flexural tests were respectively carried out after 28 days of curing in a standard curing room with a temperature of (20 ± 2) °C and a relative humidity of more than 95%.

### 2.4. Test Method

Seventeen groups of test pieces were respectively made with the above mixture ratio and the arithmetic average value of three test pieces in each group was taken as the compressive strength or flexural strength of the test pieces. The compressive strength test adopts microcomputer-controlled full-automatic pressure testing machine, model YAD-2000 (Changchun Kexin Test Instrument Co., Ltd., Changchun, China), pressure speed was 1.0 kN/s and the flexural strength test adopts DKZ-5000 electric flexural testing machine (Zhejiang Chenxin Machinery Equipment Co., Ltd., Hangzhou, China). When the difference between one of the maximum or minimum values of the three measured values and the intermediate value exceeds 15% of the intermediate value, the maximum value and the minimum value were eliminated together and the intermediate value was taken as the compressive strength or flexural strength value of the restructured specimen; If the difference between the two values and the intermediate value exceeds 15% of the intermediate value, the experimental results of this group of test pieces were invalid.

## 3. Results and Discussion

### 3.1. Test Results

The test results of 28 d compressive strength and flexural strength of basalt polypropylene fiber mortar were shown in Table 5.

### 3.2. Range Analysis 

**Figure 1:**
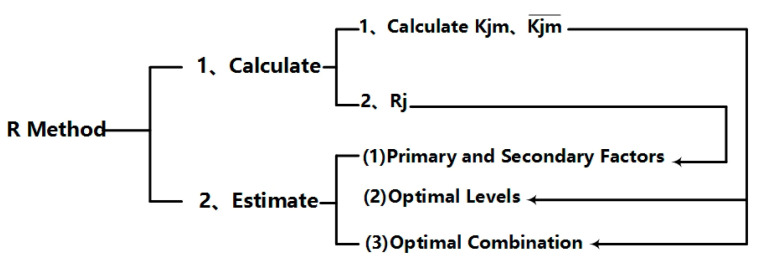
Schematic diagram of range analysis content.

The range analysis method was referred to as R method, which distributes the multi-factor and multi-level test evenly through fewer test times, so as to improve the test efficiency [27]. It consists of calculating and judging, as shown in Figure 1.

Among them, $\bar{K_{jm}}$ was the sum of test indexes *y_jm_* corresponding to m level in column factor *j*. *K_jm_* was the expected value of *y_jm_*, that is, the average value of *y_jm_*, *R_j_* was the range of the column *j* factor, same as, the difference between the maximum value and the minimum value of the index value at each level of the column *j* factor.

(1) $$K_{jm}=\sum_{n=1}^{m}y_{jm}$$

(2) $$\bar{K_{jm}}=\left(\sum_{n=1}^{m}K_{jm}\right)/m$$

(3) $$R_{j}=\max\left(\bar{K_{j1}},\bar{K_{j2}},\ldots ,\bar{K_{jm}}\right)-\min\left(\bar{K_{j1}},\bar{K_{j2}},\ldots ,\bar{K_{jm}}\right).$$

According to the size of $\bar{K_{jm}}$, the optimal level of *j* factor and the optimal level combination of each factor can be judged, that is, the optimal combination. The magnitude of *R_j_* can be used to judge the variation range of test indexes when the level of the factors in column j changes. The larger *R_j_*, the greater the influence of this factor on the test index and therefore the more important it is. Therefore, according to the size of the extremely poor *R_j_*, the primary and secondary factors can be judged.

According to the data in Table 5, range analysis of each orthogonal test was carried out to obtain the range table of orthogonal test, as shown in Table 6.

**Figure 2:**
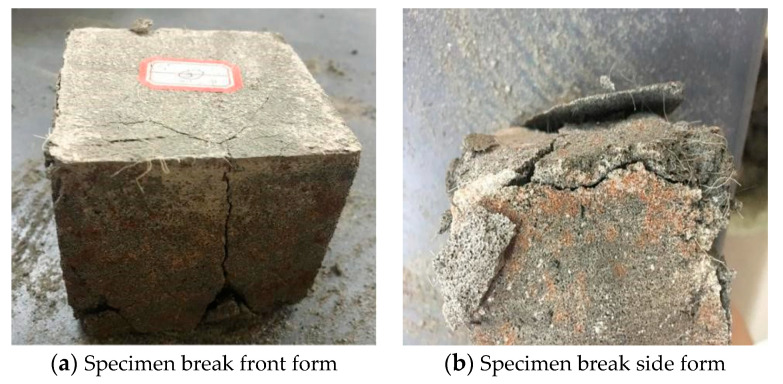
Specimen damage form.

As can be seen from the data in Table 6 that the influence of various factors on the 28 d compressive strength of mortar specimens was as follows—polypropylene fiber length > basalt fiber volume content > polypropylene fiber volume content > basalt fiber length. The influence of each factor on 28 d flexural strength was as follows—basalt fiber volume fraction > polypropylene fiber length > basalt fiber length > polypropylene fiber volume fraction. The 28 d strength effect of mortar specimens was compressive strength > flexural strength, which indicates that the fracture strength and elongation of fibers play a pulling role in the specimens. As shown in Figure 2, the specimen can still remain in its original state when it was damaged and the fiber filaments can also be clearly seen at the crack to be connected at both ends of the crack.

### 3.3. Hierarchical Analysis of Orthogonal Test

**Figure 3:**
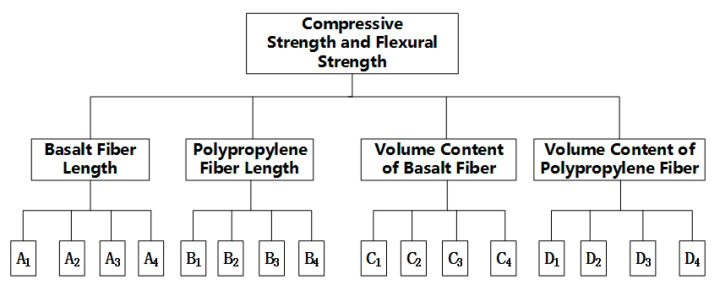
Orthogonal test Analytic Hierarchy Process (AHP) mode.

Analytic Hierarchy Process (AHP) is a method of hierarchical analysis of complex problems [28]. It combines with orthogonal experiments and analyzes indexes, factors and levels hierarchically to determine the influence weight of each factor on each index and selects the best scheme among many factors and levels. And the AHP model for the orthogonal test was illustrated in Figure 3.

As shown in Figure 3, the orthogonal test was divided into three layers. The first layer was the test index, namely compressive strength and flexural strength; the second layer was the factor (*A^(i)^*), namely basalt fiber length (*A*^(1)^), polypropylene fiber length (*A*^(2)^), basalt fiber volume content (*A*^(3)^), polypropylene fiber volume content (*A*^(4)^) and the third layer was the factor level, namely each factor was divided into four levels. From left to right, let’s call that $A_{1}^{\left(1\right)}$, $A_{2}^{\left(1\right)}$, $A_{3}^{\left(1\right)}$, $A_{4}^{\left(1\right)}$, $A_{1}^{\left(2\right)}$, $A_{2}^{\left(2\right)}$, $A_{3}^{\left(2\right)}$, $A_{4}^{\left(2\right)}$, $A_{1}^{\left(3\right)}$, $A_{2}^{\left(3\right)}$, $A_{3}^{\left(3\right)}$, $A_{4}^{\left(3\right)}$, $A_{1}^{\left(4\right)}$, $A_{2}^{\left(4\right)}$, $A_{3}^{\left(4\right)}$, $A_{4}^{\left(4\right)}$.

The sum of the test data at the level of column *j* of factor *A^(i)^* was denoted as *K_ij_*, which was called the effect of column *j* of factor *A^(i)^* on the test (*i* = 1, 2, ..., *k*; *j* = 1, 2, ..., *n_k_*). The effect weight was calculated by Equations (4)–(7).

(4) $$A=\left[\begin{array}{cccc} M_{11} & 0 & \ldots & 0\\ M_{21} & 0 & \ldots & 0\\ \ldots & \ldots & \ldots & \ldots \\ M_{n1} & 0 & \ldots & 0\\ 0 & M_{12} & \ldots & 0\\ 0 & M_{22} & \ldots & 0\\ \ldots & \ldots & \ldots & \ldots \\ 0 & M_{n2} & \ldots & 0\\ \ldots & \ldots & \ldots & \ldots \\ 0 & 0 & \ldots & M_{1k}\\ 0 & 0 & \ldots & M_{2k}\\ \ldots & \ldots & \ldots & \ldots \\ 0 & 0 & \ldots & M_{nk} \end{array}\right]$$

(5) $$S=\left[\begin{array}{cccc} 1/t_{1} & 0 & \ldots & 0\\ 0 & 1/t_{2} & \ldots & 0\\ \ldots & \ldots & \ldots & \ldots \\ 0 & 0 & \ldots & 1/t_{k} \end{array}\right]$$

(6) $$t_{j}=\sum_{j=1}^{n_{j}}M_{ij}$$

(7) $$C=\left[\begin{array}{c} \begin{array}{cccc} \frac{R_{1}}{\sum_{i=1}^{k}R_{i}} & \frac{R_{2}}{\sum_{i=1}^{k}R_{i}} & \ldots & \frac{R_{k}}{\sum_{i=1}^{k}R_{i}} \end{array} \end{array}\right]$$

*A* represents the horizontal layer which affects the test effect matrix; *C* represents the influence of factors on the experiment weight matrix. The influence weight of each factor level on the test index was calculated as: *w* = *ASC*^T^, it indicates the influence of various factors and levels on the test indexes [29].

Using this model computed the relative weighting of every factor level and the results was shown in Table 7.

As shown in Table 7, for compressive strength, the four horizontal weights of basalt fiber length were similar but A1 (6 mm) has the largest weight, B1 (3 mm) has the largest weight among the four levels of polypropylene fiber length, C4 (0.4%) has the largest weight among the four levels of basalt fiber volume content and D1 (0.1%) has the largest weight among the four levels of polypropylene fiber volume content. For flexural strength, the four horizontal weights of basalt fiber length are similar but A1 (6 mm) has the largest weight, B2 (6 mm) has the largest weight among the four levels of polypropylene fiber length, C4 (0.4%) has the largest weight among the four levels of basalt fiber volume content and D4 (0.4%) has the largest weight among the four levels of polypropylene fiber volume content. The fibers form a three-dimensional random mesh support structure in the cement matrix and the surface area per unit volume increases due to the increase of the volume content, thus reducing the spacing between the fibers and the more cement matrix wrapped on the surface of the fibers, the fibers can bear the stress generated by the volume deformation of the cement matrix.

### 3.4. Factor Index Analysis

**Figure 4:**
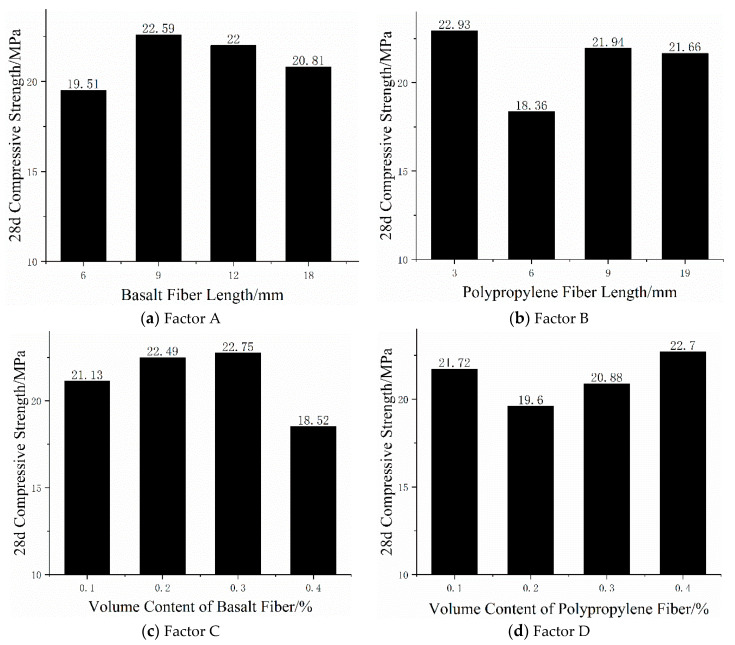
Relationship between four factors and compressive strength.

**Figure 5:**
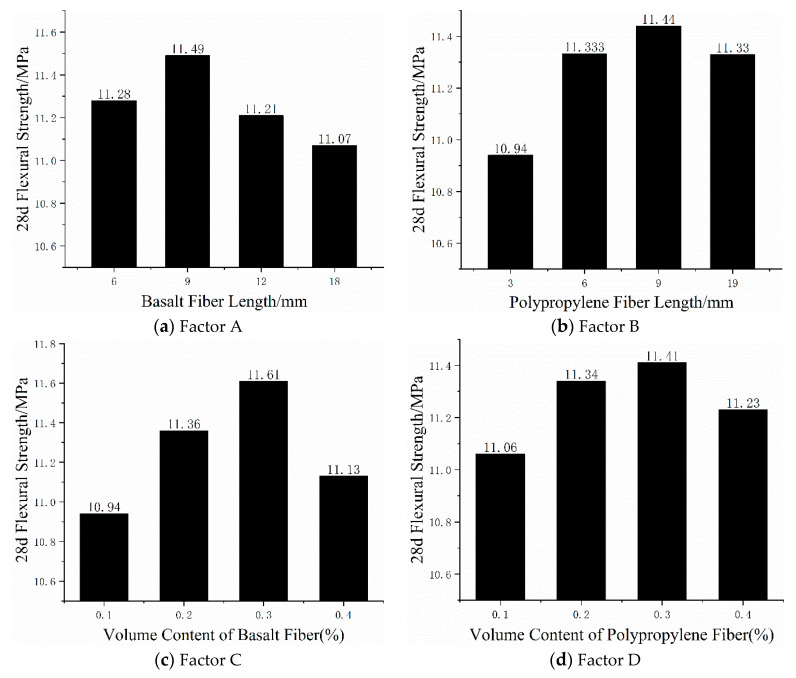
Relationship between four factors and flexural strength.

According to the orthogonal test method, the 28 d compressive strength and flexural strength investigated are called indexes. The average strength of each factor at each level can be obtained from the visual analysis table, as shown in Figure 4 and Figure 5.

As can be seen from Figure 4a, when the basalt fiber length increased from 6 to 9 mm, the compressive strength of mortar specimens increased by 15.79% but the basalt fiber length increased from 9 to 18 mm and the compressive strength of mortar specimens decreased by 7.88%. When the basalt fiber length increased from 9 to 12 mm, the compressive strength decreased by 2.61%, from 12 to 18 mm and decreased by 5.41%. As can be seen from Figure 5a, when the basalt fiber length increased from 6 to 9 mm, the flexural strength of mortar specimens increased by 1.86% but when the basalt fiber length increased from 9 to 18 mm, the flexural strength of mortar specimens decreased by 3.66%. When the basalt fiber length increased from 9 to 12 mm, the flexural strength decreased by 2.44%, from 12 to 18 mm and decreased by 1.25%. According to the above analysis, the best choice of basalt fiber length for compressive strength and flexural strength was 6–9 mm. Both compressive strength and flexural strength show a trend of increasing first and then taking effect with the increase of basalt fiber length. Analysis shows that the reason for this phenomenon was that the basalt fiber length was too short or too long will limit the crack suppression of cement mortar and cannot effectively play a bridging role in mortar. Compared with long fibers, short fibers can be more evenly dispersed in mortar and better disperse and transfer the energy of mortar in hand, so as to improve the mechanical properties of mortar.

As can be seen from Figure 4b, when the length of polypropylene fiber increased from 3 to 6 mm, the compressive strength decreased by 19.93%, from 6 to 9 mm, the compressive strength increased by 19.50%, from 9 to 19 mm and the compressive strength decreased by 1.28%. As can be seen from Figure 5b, when the length of polypropylene fiber increased from 3 to 9 mm, the flexural strength of mortar specimens increased by 4.57%, the length of polypropylene fiber increased from 3 to 6 mm, the flexural strength increased by 3.59%, the length increases from 6 to 9 mm and the flexural strength increased by 0.94%. However, the length of polypropylene fiber increased from 9 to 19 mm and the flexural strength decreased by 0.96%. From the above analysis, the best choice of polypropylene fiber length for compressive strength and flexural strength index was 6–9mm. From the analysis of the above phenomena, it can be seen that when the length of polypropylene fiber was too short, the distribution inside the mortar will be uneven, which will reduce the compactness of the mortar, thus affecting the compressive strength and flexural strength. However, excessively long polypropylene fibers tend to agglomerate in mortar and cannot normally exert its strong tensile strength, which makes some mortar ineffective, thus reducing the flexural strength of mortar.

As can be seen from Figure 4c, the compressive strength of mortar specimens increased first and then decreases with the increased of basalt fiber volume content. When the volume content of basalt fiber increased from 1% to 3%, its compressive strength increased by 7.67%. Among them, the content increased from 1% to 2%, the compressive strength increased by 6.43%, from 2% to 3% and the compressive strength increased by only 1.16%. However, when the volume content increased from 3% to 4%, the compressive strength decreased by 18.59%. As can be seen from Figure 5c, the volume content of basalt fiber increased from 1% to 3% and the flexural strength of mortar specimens increased by 6.12%. Among them, the content increased from 1% to 2%, the flexural strength increased by 3.84%, from 2% to 3% and the flexural strength increased by 2.2%. However, the volume content increased from 3% to 4%, while the flexural strength decreased by 4.13%. From the above analysis, it can be seen that the best choice of basalt fiber volume content for compressive strength and flexural strength index was 2%–3%. The compressive strength and flexural strength of fiber mortar show a trend of increasing first and then decreasing with the increase of basalt fiber length, which shows that a reasonable fiber content was conducive to the improvement of compressive strength and flexural strength and a negative hybrid effect will occur if the fiber content was too high. The reason was that basalt fiber was properly added and the fiber can be evenly dispersed in the mortar to form a little bonding force with the mortar, thus producing an effective crack resistance effect. In addition, the random support system formed by fibers and mortar bear the load together, effectively improving the stress form of cement-based materials and improving the compressive strength and flexural strength.

As can be seen from Figure 4d, the polypropylene fiber content increased from 1% to 2% and the compressive strength of mortar specimens decreased by 9.76% but the polypropylene fiber content increased from 2% to 4% and the compressive strength of mortar specimens increased by 15.82%. When the polypropylene fiber content increased from 2% to 3%, the compressive strength increased by 6.53%, from 3% to 4% and the compressive strength increased by 8.72%. When the content of polypropylene fiber was too small, the reinforcing effect of fiber in mortar will not be formed, so the compressive strength of mortar will not be greatly improved. Due to the good crack resistance ability of polypropylene fiber, the compressive strength of mortar increases gradually with the increasing content of polypropylene fiber. However, when the content of polypropylene fiber was too high, polypropylene fiber was easy to agglomerate in mortar, which makes the void inside mortar larger and the fine aggregate was unevenly distributed in mortar, resulting in the decrease of mortar compactness, thus reducing the compressive strength. As can be seen from Figure 5d, when the volume content of polypropylene fiber increased from 1% to 2%, the flexural strength increased by 2.53%, from 2% to 3%, the flexural strength increased by 0.62%, from 3% to 4% but the compressive strength decreased by 1.58%. With the increase of polypropylene fiber content, the flexural strength of mortar increases first and then decreases. The reason for this phenomenon was that polypropylene fiber has high tensile strength. The addition of polypropylene fiber will improve the brittleness and toughness of cement mortar, enhance the cohesiveness between cement mortar and aggregate and thus improve the flexural strength. The same too much fiber content was easy to agglomerate in mortar, which cannot give full play to its function of preventing crack formation and expansion, resulting in the reduction of flexural strength. From the above analysis, we know that the best choice of polypropylene fiber volume content for compressive strength and flexural strength index was 2%–3%.

### 3.5. Analysis of Variance

The range analysis method has been carried out previously but since the range analysis method cannot estimate the error in the test process and the test result determination and cannot distinguish the data fluctuation caused by the change of test conditions from the data fluctuation caused by the error, in order to make up for this shortcoming, the variance analysis method was adopted to distinguish the difference and error in the test result caused by the change of factor level. At the same time, the contribution rate analysis can further confirm the influence of factors on indicators [30]. The results of 28 d compressive strength, variance of flexural strength and contribution rate of various factors and errors were shown in Table 8 and Table 9.

From Table 8, it can be seen that basalt fiber length, polypropylene fiber length, basalt fiber volume content and polypropylene fiber volume content have significant effects on 28 d compressive strength of mortar specimens. However, polypropylene fiber length was the main factor affecting the compressive strength of mortar specimens, basalt fiber volume content was the second, followed by polypropylene fiber volume content, basalt fiber length has the smallest effect, which was consistent with the results of range analysis. At the same time, polypropylene fiber length has the greatest influence on the 28 d compressive strength and its contribution rate reaches 33.12%, which was far greater than the data fluctuation caused by errors. The volume content of basalt fiber was 32.93%, which was much larger than the data fluctuation caused by error. The contribution currency of polypropylene fiber volume content and basalt fiber length is also larger than the data fluctuation caused by errors but the contribution ratio of basalt fiber length was the smallest among the four.

From Table 9, it can be seen that the influence of various factors on the flexural strength at 28 d was as follows—the volume content of polypropylene fiber was the largest, the length of polypropylene fiber was the second, the length of basalt fiber was the third and the volume content of polypropylene fiber was the smallest, which is consistent with the result of range analysis. The influence of four factors on flexural strength was shown as follows—volume content of polypropylene fiber, polypropylene fiber length and basalt fiber length were all significant, while volume content of polypropylene fiber was influential. In the mixing process, the contribution ratio of polypropylene fiber volume content was 32.08% at the maximum, which was 32 times of the error. The contribution ratio of polypropylene fiber length was 26.18%, which was 26 times of the error contribution ratio. The contribution ratios of basalt fiber length and polypropylene fiber volume content were 15.8% and 9.72% respectively, which were 15 and 9 times of the error contribution ratio.

### 3.6. Efficacy Coefficient Analysis

The efficacy coefficient method can comprehensively evaluate multi-objective problems. According to the orthogonal test results, the maximum and minimum values of 28 d compressive strength value and flexural strength value were selected as the satisfactory value and impermissible value of the efficacy coefficient method index evaluation system [31]. The specific values were shown in Table 10.

According to the satisfactory value and impermissible value of each index, the efficiency coefficient value of each index was calculated and the formula is following:

(8) $$d_{i}=\frac{Actual\;value-\mathrm{Impermissible}\;\mathrm{value}\;}{Satisfaction\;value-\mathrm{Impermissible}\;value}\times 40+60.$$

The results are shown in Table 11.

According to the calculation result of efficacy coefficient, the best performance for the compressive strength was specimen 12 and its ratio combination was A4B1C2D4; for the flexural strength, the best performance was specimen 14 and its ratio combination was A2B2C4D4. According to the comparison of the total efficacy coefficient values, the total efficacy coefficient score of test piece 14 was the highest, which was 95.38. Therefore, combining the three strength indexes, the best matching combination was A2B2C4D4. Compared with the blank test piece, the compressive strength of test piece 14 was increased by 27.04% and the flexural strength was increased by 49%.

### 3.7. The Action Mechanism of Fiber 

An appropriate amount of polypropylene fiber was mixed into cement mortar to form a uniform three-dimensional random support system, which can reduce segregation and bleeding of the mixture and the bridging effect of polypropylene fiber can also limit further crack propagation during cracking process. When the fiber content is too large, the fluidity of the mixture will decrease rapidly and the matrix will be hard to be compacted. Basalt fibers form a spatial grid structure inside the mixture structure, which plays a role on supporting aggregates, reducing segregation and bleeding of the mixture and can also inhibit the propagation of microcracks in the matrix through bridging. Due to the small diameter of basalt fibers, the fibers are easy to intertwine with each other during the dispersion process in cement mortar, resulting in “agglomeration”.

Due to the large gap in diameter and elastic modulus between polypropylene fiber and basalt fiber, the two fibers can form a “gradation” when they were reasonably matched, thus forming a more uniform and dense spatial network structure in the mixture, weakening the unfavorable conditions in the mixing and forming process and limiting the generation and development of shrinkage microcracks and plastic settlement cracks in the hardening process. In the loading process, basalt fiber firstly limited the propagation of microcracks. As microcracks fuse and coalesce into larger cracks, the basalt was gradually pulled out. In this process, polypropylene fiber also gradually played the bridging and bearing role, which limited the further growth of the crack. Finally, the overall performance of the cement matrix was improved by the combination of the two fiber and produce a positive hybrid effect.

The simultaneous incorporation of the two fibers into the cement matrix will increase the total amount of fibers, which may lead to poor workability of the mixture, thus reducing the compactness of the cement matrix. In addition, if the mixing content of polypropylene fiber and basalt fiber was unreasonable, the two fibers will affect each other in the mixing process, resulting in uneven fiber dispersion or intertwining, agglomeration and formation of more weak areas, thus greatly reducing the compactness of cement matrix, which will degrade its various mechanical performance indexes and produce negative hybrid effect.

### 3.8. Microscopic Mechanism Analysis

**Figure 6:**
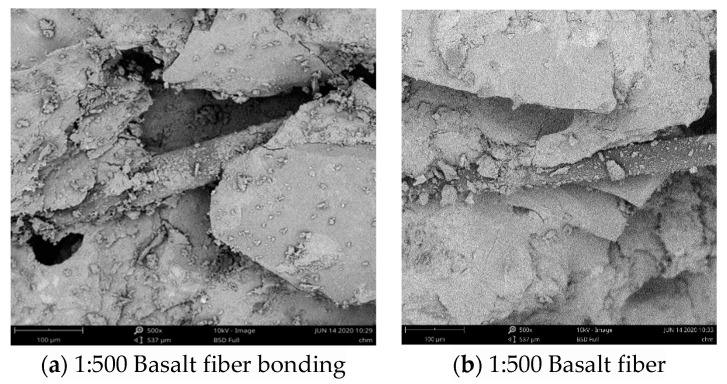
SEM of basalt fiber mortar.

**Figure 7:**
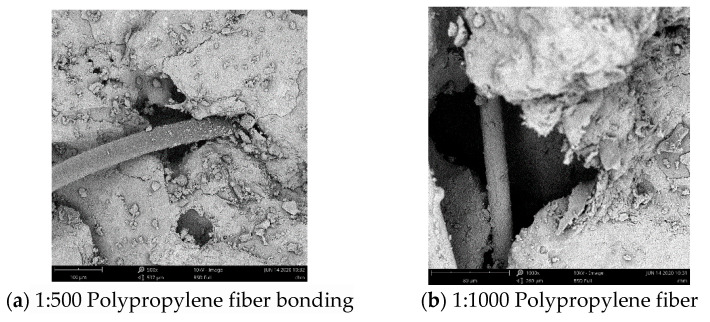
SEM of polypropylene fiber mortar.

This paper summarizes the previous research results and analyzes the reasons for the strength of fiber-reinforced cement-based materials from the microstructure with the aid of Phenom scanning electron microscope (SEM). Figure 6 and Figure 7 respectively reflect the bonding and internal porosity of basalt fiber and polypropylene fiber with cement-based materials under different magnification.

As can be seen from Figure 6, basalt fiber spans the cracks in the matrix, combines with slurry with monofilament finer than polypropylene, has a good combination form with mortar and the surface was wrapped with a large amount of cement colloid to make up the pores at the bonding place, further inhibiting the generation and expansion of microcracks and improving the mechanical properties of mortar and the crack resistance of fiber mortar. As can be seen from Figure 7, polypropylene fibers were uniformly distributed in the cement-based material in a monofilament shape, which plays a bridging role and anchoring role on the material structure. Discontinuities with larger pores and microcracks in the cement-based material were often weak in strength. Polypropylene fibers can be used as connecting materials to bridge discontinuities at both ends, reducing the occurrence of microcracks and enhancing the strength of the cement-based material. Because basalt fiber and polypropylene fiber were high elastic modulus fiber and low elastic modulus fiber respectively, the two fibers play the role of inhibiting microcrack propagation at different levels. When the tensile stress was low, micro cracks appear in the cement-based material and polypropylene fibers vertically distributed in the stress section bear the tensile stress transmitted by the cracks. With the increase of the tensile stress, polypropylene fibers were pulled or pulled out and become invalid. At this time, the tensile stress between cracks was borne by basalt fibers, the crack propagation speed decreases and the strength of fiber mortar was improved.

## 4. Conclusions

After systematic analysis of basalt-polypropylene fiber mortar, the following conclusions were obtained:

- Through the analysis of orthogonal test results, it was found that the influence of each factors on 28 d compressive strength was the polypropylene fiber length > basalt fiber volume content > polypropylene fiber volume content > basalt fiber length; The effect on 28 d flexural strength was the basalt fiber volume content > polypropylene fiber length > basalt fiber length > polypropylene fiber volume content.
- When the basalt fiber length increased from 6 to 9 mm, the compressive strength of mortar specimens increased by 15.79% but when the basalt fiber length increased from 9 to 18 mm, the compressive strength of mortar specimens decreased by 7.88%. When the basalt fiber length increased from 6 to 9 mm, the flexural strength of mortar specimens increased by 1.86% but when the basalt fiber length increased from 9 to 18 mm, the flexural strength of mortar specimens decreased by 3.66%. Therefore, the best selection range of basalt fiber length can be determined to be 6–9 mm.
- When the length of polypropylene fiber increased from 3 to 6 mm, the compressive strength decreased by 19.93%, the length increased from 6 to 9 mm, the compressive strength increased by 19.50%, the length increased from 9 to 19 mm and the compressive strength decreased by 1.28%. When the length of polypropylene fiber increased from 3 to 9 mm, the flexural strength of mortar specimens increased by 4.57% but the length of polypropylene fiber increased from 9 to 19 mm and the flexural strength decreased by 0.96%. Thus, the optimal selection range of the length of polypropylene fiber was determined to be 6–9mm.
- When the volume content of basalt fiber increased from 1% to 3%, its compressive strength increased by 7.67% and the content of basalt fiber increased from 3% to 4%, its compressive strength decreased by 18.59%. The volume content of basalt fiber increased from 1% to 3%, the flexural strength of mortar specimens increased by 6.12%, while the content of basalt fiber increased by 4% but the flexural strength decreased by 4.13%. Thus, the optimal selection range of the content of basalt fiber was determined to be 2%–3%.
- The compressive strength of mortar specimens decreased by 9.76% when the polypropylene fiber content increased from 1% to 2% but the compressive strength of mortar specimens increased by 15.82% when the polypropylene fiber content increased from 2% to 4%. When the volume content of polypropylene fiber was increased by 2% from 1%, the flexural strength is increased by 2.53%, from 2% to 3%, the flexural strength is increased by 0.62%, from 3% to 4% but the compressive strength was decreased by 1.58%, the best selection range of the volume content of polypropylene fiber can be determined to be 2%–3%.
- The best combination of compressive strength was the basalt fiber length of 18 mm, polypropylene fiber length of 3 mm, basalt fiber content of 0.2% and polypropylene fiber content of 0.4. The best combination of flexural strength was the basalt fiber length of 9 mm, the polypropylene fiber length of 6mm, the basalt fiber content of 0.4% and the polypropylene fiber content of 0.4. According to the efficiency coefficient value, the best combination of comprehensive strength was that the basalt fiber length was 9 mm, the polypropylene fiber length was 6mm, the basalt fiber content was 0.4% and the polypropylene fiber content was 0.4%.

## Figures and Tables

**Table 1:** Physical and Mechanical Properties of Basalt Fiber.

Fiber Diameter/um	Fiber Type	Density/g·cm^−3^	Elongation at Break/%	Elastic Modulus/GPa	Tensile Strength/MPa
7–15	Short cut	2.63–2.65	3.1	91–110	3000–4800

**Table 2:** Physical and Mechanical Properties of Polypropylene Fiber.

Fiber Diameter/um	Fiber Type	Density/g·cm^−^^3^	Fracture Elongation /%	Young’s Modulus/GPa	Tensile Strength/MPa
31	monofilament	0.91	30	≥3.5	≥460

**Table 3:** Factors-Level Table.

	Divisor	Basalt Fiber Length/mm	Polypropylene Fiber Length/mm	Basalt Fiber Volume Content/%	Polypropylene Fiber Volume Content/%
Level					
1		6	3	0.1	0.1
2		9	6	0.2	0.2
3		12	9	0.3	0.3
4		18	19	0.4	0.4

**Table 4:** Testing Arrangement.

	Divisior	Basalt Fiber Length/mm	Polypropylene Fiber Length/mm	Basalt Fiber Volume Content/%	Polypropylene Fiber Volume Content/%
Test Number					
1		6	6	0.3	0.3
2		9	19	0.1	0.2
3		12	19	0.3	0.4
4		18	6	0.1	0.1
5		6	9	0.1	0.4
6		9	3	0.3	0.1
7		12	3	0.1	0.3
8		18	9	0.3	0.2
9		6	3	0.4	0.2
10		9	9	0.2	0.3
11		12	9	0.4	0.1
12		18	3	0.2	0.4
13		6	19	0.2	0.1
14		9	6	0.4	0.4
15		12	6	0.2	0.2
16		18	19	0.4	0.3

**Table 5:** Orthogonal Test Results.

	Item Test Number	Basalt Fiber Length/mm	Polypropylene Fiber Length/mm	Basalt Fiber Volume Content/%	Polypropylene Fiber Volume Content/%	Compressive Strength/MPa	Flexural Strength/MPa
Test Number							
0		0	0	0	0	18.73	8.66
1		6	6	0.3	0.3	16.05	11.50
2		9	19	0.1	0.2	20.17	10.08
3		12	19	0.3	0.4	18.71	10.32
4		18	6	0.1	0.1	20.18	10.76
5		6	9	0.1	0.4	23.30	11.33
6		9	3	0.3	0.1	19.58	10.57
7		12	3	0.1	0.3	20.87	10.59
8		18	9	0.3	0.2	19.76	11.14
9		6	3	0.4	0.2	25.14	11.62
10		9	9	0.2	0.3	19.54	11.45
11		12	9	0.4	0.1	25.18	11.41
12		18	3	0.2	0.4	26.12	10.98
13		6	19	0.2	0.1	25.85	11.51
14		9	6	0.4	0.4	18.76	12.01
15		12	6	0.2	0.2	18.46	11.50
16		18	19	0.4	0.3	21.92	11.39

**Table 6:** Orthogonal Range Analysis of Various Factors Range Analysis.

Factor Categories	28 d Compressive Strength/MPa	28d Flexural Strength/MPa
Factor A	3.07	0.42
Factor B	4.57	0.50
Factor C	3.97	0.67
Factor D	3.10	0.35

**Table 7:** Weight of Each Factors Influence on Each Indicator.

Factors Index	Compressive Strength (Weight)	Flexural Strength (Weight)
A1	0.057	0.060
A2	0.049	0.059
A3	0.052	0.058
A4	0.055	0.057
B1	0.086	0.068
B2	0.069	0.071
B3	0.082	0.070
B4	0.081	0.070
C1	0.069	0.091
C2	0.073	0.094
C3	0.060	0.092
C4	0.074	0.096
D1	0.058	0.029
D2	0.053	0.030
D3	0.050	0.029
D4	0.055	0.031

**Table 8:** Variance Analysis and Contribution Rate of Factors and Errors to Compressive Strength.

Factor	Sum of Squares of Deviations	Degree of Freedom	Mean Square	F Value	Critical Value	Statistical Significance	Pure Sum of Squares	Contribution Rate/%
A	22.869	3	7.623	30.428	F0.1(3,3) = 5.39	**	22.116	16.03
B	46.447	3	15.482	61.798	F0.01(3,3) = 29.46	**	45.694	33.12
C	46.189	3	15.396	61.455	F0.05(3,3) = 9.28	**	45.436	32.93
D	29.049	3	9.683	38.649		**	28.296	20.51
Error	0.752	3	0.251					0.1
Sum	137.968	15						

Note: ⊙ means affected; * means significant; ** means highly significant.

**Table 9:** Variance Analysis and Contribution Rate of Factors and Errors to Flexural Strength.

Factor	Sum of Squares of Deviations	Degree of Freedom	Mean Square	F Value	Critical Value	Statistical Significance	Pure Sum of Squares	Contribution Rate/%
A	0.400	3	0.133	9.765	F0.1(3,3) = 5.39	⊙	0.358	15.80
B	0.632	3	0.211	15.425	F0.01(3,3) = 29.46	*	0.590	26.18
C	0.765	3	0.255	18.662	F0.05(3,3) = 9.28	*	0.723	32.08
D	0.261	3	0.087	6.380		⊙	0.219	9.72
Error	0.041	3	0.014					0.1
Sum	2.254	15						

Note: ⊙ means affected; * means significant; ** means highly significant.

**Table 10:** Satisfactory and Disallowed Values for Each Indicator.

Value Tables	28 d Compressive Strength/MPa	28 d Flexural Strength/MPa
Satisfaction value	26.12	12.01
Impermissible value	16.05	10.57

**Table 11:** Efficacy Coefficient Analysis Result.

Number	Efficacy Indicators/MPa		Efficacy Coefficient		Total Efficacy Coefficient
	Compressive Strength	Flexural Strength	Compressive Strength	Flexural Strength	
1	16.05	11.50	60.00	85.83	72.92
2	20.17	10.08	76.37	74.17	75.27
3	18.71	10.32	70.57	80.83	75.70
4	20.18	10.76	76.41	65.28	70.84
5	23.30	11.33	88.80	81.11	84.95
6	19.58	10.57	74.02	60.00	67.01
7	20.87	10.59	79.15	60.56	69.85
8	19.76	11.14	74.74	75.83	75.29
9	25.14	11.62	96.11	89.17	92.64
10	19.54	11.45	73.86	84.44	79.15
11	25.18	11.41	96.27	83.33	89.80
12	26.12	10.98	100.00	71.39	85.70
13	25.85	11.51	98.93	86.11	92.52
14	18.76	12.01	90.76	100.00	95.38
15	18.46	11.50	69.57	85.83	77.70
16	21.92	11.39	83.32	82.78	83.05
